# Women and leprosy: interferences and experiences*

**DOI:** 10.1590/1518-8345.4347.3419

**Published:** 2021-07-02

**Authors:** Marcela Gonçalves, Karen da Silva Santos, Simone Santana da Silva, Thalita Caroline Cardoso Marcussi, Kisa Valladão Carvalho, Cinira Magali Fortuna

**Affiliations:** 1 Universidade de São Paulo, Escola de Enfermagem de Ribeirão Preto, PAHO/WHO Collaborating Centre for Nursing Research Development, Ribeirão Preto, SP, Brazil.; 2 Scholarship holder at the Conselho Nacional de Desenvolvimento Científico e Tecnológico (CNPq), Brazil.; 3 Universidade do Estado da Bahia, Campus VII, Senhor do Bonfim, Bahia, Brazil.; 4 Scholarship holder at the Fundação de Amparo à Pesquisa do Estado de São Paulo (FAPESP), Brazil.

**Keywords:** Women, Leprosy, Social Vulnerability, Public Health, Work, Health Personnel, Mujeres, Lepra, Vulnerabilidad Social, Salud Publica, Trabajo, Personal de Salud, Mulheres, Hanseníase, Vulnerabilidade Social, Saúde Pública, Trabalho, Pessoal de Saúde

## Abstract

**Objective:**

to know the interferences of leprosy in women's lives and how they reinvent themselves in coping with the disease.

**Method:**

a descriptive study with a qualitative approach. The theoretical-methodological framework adopts an approximation to the cartographic method and some concepts of schizoanalysis, which were used to analyze the data. The tools used to produce the data were the interview and the logbook. The interviews were conducted from July to November 2019, at the participants' homes.

**Results:**

the group consisted of nine women. To display the data, we were inspired by Deleuze's ideas about difference and repetition. The results were organized in three thematic axes that address the lives of these women affected by leprosy, which accompany concerns, anxieties and worries about the effects of the disease. The transformations in the female body, the financial maintenance itself due to the comorbidities caused by leprosy and its difficulties in guaranteeing rights are elements strongly pointed out by women.

**Conclusion:**

there is overlap and interference of the female condition in a patriarchal society that still accompanies it. We bet on the strength of becoming-a-woman and the need to consider them in their singularities and in their context for producing care permeated by meetings of the affirmation of the power of life.

## Resumo

**Objetivo:**

conocer las interferencias de la enfermedad de Hansen en la vida de las mujeres y cómo se reinventan en el afrontamiento de la enfermedad.

**Método:**

estudio de abordaje cualitativo. El marco teórico-metodológico adopta un acercamiento al método cartográfico y algunos conceptos de esquizoanálisis, que fueron utilizados para analizar los datos. Las herramientas utilizadas para producir los datos fueron la entrevista y el libro de registro. Las entrevistas se realizaron de julio a noviembre de 2019, en los domicilios de las participantes.

**Resultados:**

el grupo estuvo compuesto por nueve mujeres. Para presentar los datos, nos inspiramos en las ideas de Deleuze sobre la diferencia y la repetición. Los resultados se organizaron en tres ejes temáticos que abordan la vida de estas mujeres afectadas por la enfermedad de Hansen, que acompañan inquietudes, angustias y preocupaciones sobre los efectos de la enfermedad. Los cambios en el cuerpo femenino, la manutención económica propiamente dicha debido a las comorbilidades provocadas por la enfermedad de Hansen y sus dificultades para garantizar los derechos son elementos fuertemente señalados por las mujeres.

**Conclusión:**

hay una superposición e interferencia de la condición femenina en una sociedad patriarcal que aún la acompaña. Apostamos por la fuerza del devenir-mujer y la necesidad de considerarla en sus singularidades y en su contexto para la producción de cuidados forjados por encuentros para afirmar el poder de la vida.

## Abstract (pt)

**Objetivo:**

conhecer as interferências da hanseníase na vida das mulheres e como elas se reinventam no enfrentamento da doença.

**Método:**

estudo de abordagem qualitativa. O referencial teórico-metodológico adota uma aproximação ao método cartográfico e alguns conceitos da esquizoanálise, que foram utilizados para analisar os dados. As ferramentas utilizadas para produção dos dados foram a entrevista e o diário de bordo. As entrevistas foram realizadas no período de julho a novembro de 2019, no domicílio das participantes.

**Resultados:**

o grupo foi composto por nove mulheres. Para apresentar os dados, nos inspiramos nas ideias de Deleuze relativas à diferença e repetição. Os resultados foram organizados em três eixos temáticos que abordam a vida dessas mulheres afetadas pela hanseníase, os quais acompanham inquietações, angústias e preocupações frente aos efeitos da doença. As alterações no corpo feminino, a própria manutenção financeira devido às comorbidades causadas pela hanseníase e as suas dificuldades nas garantias de direitos são elementos fortemente apontados pelas mulheres.

**Conclusão:**

há sobreposição e interferência da condição feminina em uma sociedade patriarcal que ainda a acompanha. Apostamos na força do devir-mulher e na necessidade de considerá-la em suas singularidades e em seu contexto para a produção do cuidado permeado por encontros de afirmação da potência da vida.

## Introduction

Leprosy is an infectious disease caused by Mycobacterium leprae, which, if left untreated, can lead to disabilities^1^. It is an ancient disease, still very stigmatized in society^2^. It is on the list of neglected diseases, which affects the population of the most disadvantaged regions, and is included in the international agenda of the global commitments made by the United Nations (UN)^3^. We know that the neglected diseases, in addition to being a public health problem, have low investments in the discovery of new treatments, in research and in the necessary measures for its control.

Brazil is a country with a high burden for the disease and ranks second in the list of countries with the highest number of cases in the world^4^. Brazil presented a 30% reduction in the number of case detections, from 40,100 in 2007 to 25,200 in 2016^5^. Despite the numbers, the fight is still arduous, since its challenges are not only restricted to the biological sphere, as the cure has been achieved with multidrug therapy. It is also important to consider comprehensive care for people affected by leprosy, involving social and cultural aspects in the quest to overcome the present stigma^6^. 

Individuals are subjective beings, built in the political, economic, historical and cultural circumstances. In this sense, the subjectivity of women with leprosy is marked by a history of inequality and violence. In the context of Brazilian women, leprosy causes physical transformations due to the clinical manifestations of the disease, transforms the color of the skin through polychemotherapy, brings psychological impacts by the diagnosis, given that, for being a woman and having the functions that are assigned to her socially, everything gets exacerbated. 

The person affected by leprosy experiences a so-called psychological vulnerability, not only due to the stigma associated with the disease, but also due to the consequences of the disease and the treatment itself. A research study^7^ states that, with the use of drugs and with leprosy reactions, people affected by the disease can develop transformations in the perception of their body image. They also point out these people's difficulties in continuing daily life and work activities, which can lead them to isolate themselves socially. In the same direction, another survey^8^ refers to women with leprosy and the impacts produced by transformations in their physical body, affecting self-esteem and affective relationships.

Thus, the diagnosis of leprosy brings specificities for women, after all, in addition to being accompanied by the stigma inherent in the disease, it is added to the gender standards socially imposed on this social group.

According to another study^9^, the situation of social vulnerability occurs through the alteration of the potential for reaction to difficult situations. By accepting that people are vulnerable, the possibility of placing them under guardianship emerges, thinking that they do not know how to take care of themselves. Therefore, it is essential that the health team that provides care to women with leprosy is prepared to identify and understand the specificities that involve this condition. It is necessary to know the way in which this assistance is being provided, to listen to the person who receives the assistance and to know their strengths and potential.

The present production contributes towards knowing the ways of life of women who live with leprosy, in valuing the gender issues that permeate their routine and with adversities arising from financial and labor problems (how they deal, how they managed to reinvent themselves on their journey and continue life).

With this, it is expected to encourage reflections to professionals and future health professionals for such complexities, which need attention, to provide comprehensive care to women affected by leprosy, who suffer from society's demand of being a woman, added to the weight of the diagnosis^2^^,^ 10 ^-^ 11. Thus, this study aims to understand the interferences of leprosy in the lives of women and how they reinvent themselves in coping with the disease. 

## Method

This is a descriptive study with a qualitative approach. The theoretical methodological framework adopted was an approximation to the cartographic method and some concepts of schizoanalysis by Deleuze and Guattari, such as, for example, affection and power. The tools used to produce the data were the interview and the logbook.

The cartography was presented by Deleuze as a method and way for producing knowledge. Here, we will bring social cartography, which involves movements, relationships, power games, confrontation between forces, struggles, statements, practices of resistance and freedom^12^. Cartography is a means of creating research and the researcher's encounter with his field. In this case, the researcher will be producing knowledge considering their perceptions, paying attention to the affections and sensations produced when they live the research. These encounters produce and undo meanings^13^. 

In cartographic research, annotations are used, which are prepared after the activities involving data production^14^. We will call these notes a logbook. The registration of the construction of the research must be done not to know what is researched but, above all, to witness the research process^15^. From the first contact with the Health Unit, the diary began with the record of the researcher's impressions, feelings and perceptions. Particular attention was paid to the ways in which the service works, to the relationships that professionals established with women and to the affections triggered in the meetings with the participants and with the field. In addition to writing, audio was used, which allowed recording at different times, that is, before starting data production, during and after it. These records were considered in the analysis, bringing the possibility to contextualize and reflect on the research process. In this way, it was possible to exercise the cartography of affections and encounters that lead women undergoing leprosy treatment to create coping strategies for adversity. Also in relation to the approach to the cartographic method, it was possible to find and experience this power in the dialog between the participant and the researcher.

Data production took place from July to November 2019, in a municipality in the inland of the state of São Paulo, in a referral center for specialties that offers leprosy treatment. It is important to note that, in the municipality involved, there is also another specialized outpatient clinic linked to a quaternary level hospital. This outpatient clinic was not a field of investigation because it also serves the region, with several municipalities making up the majority of its target population and, thus, the research was restricted to the chosen municipality that is considered endemic in leprosy.

At first, the project was presented to the manager of the health unit and she talked to the nursing team, explaining the presence of the researcher in the service and asking them to guide her as to the dates and times of the women's service. With the collaboration of the team, the researcher developed a strategy to approach possible participants at the end of the consultation or care in the service. At that moment, the objective of the research, the risks, benefits, and the interview were jointly presented to the women undergoing treatment. 

Data production took place through interviews with approaches to cartographic management, with questions in accordance with the research objective. They were carried out with women under leprosy treatment who agreed to participate, being able to choose the date, place and time. All of them chose their homes as the location of the interview because there were already other tasks scheduled after care in the health unit.

The mean duration of the interviews was 1 hour and 20 minutes, they were audio-recorded and later on transcribed for analysis. The person who conducted the interviews was one of the authors, a nurse who, at the time of data production, was pursuing graduate studies at the master's level and had already carried out qualitative research studies with interviews.

Nineteen women were invited to participate in the research, with acceptance and participation of nine; two accepted but were not found at their homes, another four women gave up, and four refused. They justified their non-participation due to lack of time and for not feeling comfortable talking to the researcher. As for the inclusion and exclusion criteria, the method used does not provide for this way of dealing with the participants, but we emphasize that we interviewed only women over 18 years old. Likewise, for surveys in cartographic mode, it is the meetings and their intensity that determine the number of participants, having as a reference the possibility of having answered the research objectives. No new interviews were conducted because they were considered sufficient in the analytical process.

After the transcription of the interviews, a careful reading was carried out with the modulation and intonations of the participant's voice (such as pauses, moments of transformation in the pitch of the voice and the perceived affections). On top of these markings, a second reading was made with emphasis on the parts of the interview that affected the researchers and that indicated thematic convergences in line with the objectives and, thus, analytical axes were constructed. The records of the interviews, as they were reread and analyzed, were enriched with the notes from the researcher's logbook. Qualitative research studies have reflexivity as reliability of data, producing changes in the researchers and in their initial perspectives. In this investigation, this process was conducted by the production of the logbook, by discussions of the findings with the research group, in the qualification and defense exam of the master's degree.

In order to keep confidentiality in relation to the names of the participants, these were replaced by the names of famous women in history, namely: Enedina Alves Marques, Raimunda Putani, Bertha Lutz, Maria da Penha, Chiquinha Gonzaga, Cora Coralina, Maria Quitéria, Anna Neri, Dandara Palmares. Affections perceived in the interview and which were described in the logbook were also added to some speech fragments. 

The research was submitted to the Research Ethics Committee and approved through the CAAE 07625519.4.0000.5393 protocol. The Free and Informed Consent Form was signed after being read and appreciated by the participants, prior to the interviews.

## Results

The group was composed by nine women, aged between 30 and 62 years old. Four women identified themselves as married, one separated, one divorced, two in a stable relationship and one single. Regarding the profession, three were away from work, one retired, one was a housewife, one a cleaning lady, one an epilator, one a seamstress and one a day care worker. Two women had completed elementary school, two had incomplete elementary school, four had completed high school and only one had completed higher education.

To present the data, we were inspired by Deleuze's ideas about difference and repetition. This philosophy shows us that, what is known is repetition, and what is contradicted is the difference^16^. We seek a non-Manichean reading, with a view to monitoring the processes experienced by women with leprosy and herein retold. These are situations related to the female condition, at the same time that new forms of reinvention and resistance are triggered every day.

The results were therefore organized into three axes: Women with leprosy: Crossroads between the Skin, the Shape and the Feminine; Earning a living: (Un)achievable work; State Violence in the Professional Practices.

### 1. Women with leprosy: Crossroad between the Skin, the Shape and the Feminine

With leprosy and its treatment, skin transformations are predicted. Such transformations are strongly cited by the interviewees and understood in different ways. Furthermore, it is important to pay attention to the fact that such transformations do not only touch the body, but concern the feminine way of presenting themselves and the relationships with the body. They also give visibility to the operation of discriminatory systems, including in relation to skin color, in their production of significant differences in cultural and material status. 

*(...) there,* [name of the professional] *told me, she played* - lowered the voice - *she's dark, right?,* - speaking normally - *she said: You'll become almost as dark as I am, laughs, then I said* - lowered the voice - *there's no problem (...)* (Dandara, shy); *Then, there's a serious problem too, I got black, I'm not that color* - pause - *the drugs left me black (...)* (Maria Quitéria, incisive).

However, what is seen as something negative for some, differently, for others is seen as something good.

*(...) and I was pretty much clear and the leprosy treatment also makes you darker, with a more beautiful color I think* laughs *gave a* - raises the voice - *tanned* laughs (Chiquinha); *(...) there is a drug, which still dyes the skin (...) and everyone thinks I'm tanned from the beach, understood?* laughs *(...)* (Raimunda).

Another aspect mentioned by the interviewees ratifies questions about the influence of the consumer society, which values a body that resembles those exposed and evaluated as "desirable". This, in our society, it leads its owner to have greater satisfaction and high self-esteem. The vain woman is repeated in the speeches. 

*(...) I liked the short very much (...) And, then, till then I stopped using...* - pause - *visits came home, then I'd put on a pair of pants (...)* - lowers the voice - *and I was a little bit dull (...)* (Bertha); *(...) I stopped taking care of myself, (...) I didn't do any eyebrows anymore, hair I don't have the courage to paint, the nails I don't feel like caring anymore, then* - raises the voice - *I stopped caring about my looks (...)* (Anna, resigned); *(...) all my hair fell out, I had to put on a hair extension. (...)* - lowers the voice - *this here is almost all hair extension (...)* - returns to the tone of voice - *there is a colleague of mine (...) she fixed the hair extension and put it on me (...)* (Cora).

### 2. Earning a Living: (Un)achievable work

Women with leprosy seek financial assistance from beneficiary bodies to continue their lives. In their speeches, women repeatedly show their concern with retirement in old age or with the maintenance of some financial gain, given the limitations presented by the disease.

*(...) I filed for INSS (...) the lawyer filed an appeal and to conduct an expert examination through the forum (...) when I did, the expert denied my case. (...) Then, my lawyer appealed (...) I was paid for7 years, my benefit was cut now in 2017 (...)* - lowers the voice - *I appealed several times,* - returns to the tone of voice - *and as I'm not working, how will I pay the INSS (...)* (Dandara); *(...) I think I'll get this new law* (retirement) *(...) because I'm going to be 57. So, I think I'll get it. (...)* - lowers the voice - *But it's good...* - returns to the tone of voice - *May the Lord give me strength, working.* (Maria da Penha).

Informal domestic work is also a concern.

*(...) I get cleaning works* - lowers the voice - *to do. (...)* - returns to the tone of voice - *my service here at home,* - lowers the voice - *I do it myself. Not even if it hurts, you must keep on going. (...)* - returns to the tone of voice - *the orthopedic doctor said that I couldn't work (...) I was reading, leprosy gives the right* - pause - *to sickness or retirement aid (...) I've contributed many years to the INPS, but at the moment I don't have a registered labor booklet -* lowers the voice. *(...)* (Anna).

Women report loss of labor, moral and human rights.

*(...) the day I was going to take the dose sometimes I wasn't going to work (...) I was a little unwell (...) it happened that one day they fired me* - raises the voice. - returns to the tone of voice - *they never claimed that it was due to some certificate (...) I was amazed,* - raises the voice - *I cried* (nervous laughs) *(...) Did I do anything wrong? But... no. (...)* - lowers the voice - *so, I just think it was because of the disease (...)* (Bertha, sad); *(...) when they found out, that there's no way of not finding out (...) I go to the doctor about three times a week (...) there was a lesion on my skin (...) I was away for 15 days. When I came back, they demanded another exam, as if it was an admission testing (...) the occupational physician refused to return. (...) I'm away without getting anything. I'm waiting for the government,* - raises the voice - *I'm registered, I'm still there (...)* - returns to the tone of voice - *without getting a penny. (...) I don't know...*- pause - lowers the voice - *what I'm going to do with my life (...)* (Cora).

Women also received other means to assist in maintaining the home.

*Sometimes* - pause - *I have help from the social worker (...)* - lowers the voice - *it's also some help there.* (Enedina); *(...) I receive the basket* (the baskets intended for the tuberculosis program of the municipality that are left are for people who are being treated for leprosy) *from there, right? And my kids get the pension, and my mom and dad help me, and I do a little something or other, you know?* - lowers the voice - *it's not easy* (Cora).

One of the women reports necessary adaptations to the domestic service, due to leprosy.

*(...)* - lowers the voice - *Polishing a pot, no more.* - returns to the tone of the voice - *Today, just passing a kitchen loofah (...) I stopped doing this, you know, like... with a lot of* - raises the voice - *force* - returns to the tone of voice - *the service. So, I do lighter. But I can still do* (Anna).

### 3. State Violence in the Professional Practices

When rights are violated, the search for help is also unattainable. The existence of nuances of power articulated to the State cuts across the entire social structure and is present in society, including the interviewed social group. For example, such nuances are observed in the first moment that follows.

*(...) talking to the doctor (...) she said* - lowers the voice - *wow, but it's going to give you such a headache (...)* - returns to the tone of voice - *I will tell you, but you own your life (...) if I were you* - lowers the voice - *I wouldn't move it, it's not worth it, you know? (...)* - returns to the tone of voice - *why don't you get sick aid? (...)* - raises the voice - *And so I did, you know? And it worked, thanks God. Thanks God, it worked... there, I didn't even open a process, nor did I, you know...* (Bertha, satisfied).

One of the interviewees reports that she suffered domestic violence for a long time, being physical and psychological aggressions from her then husband, seeking support for her decision to report him.

*(...) there is a sister* (from the church) *who is a military police officer (...) I said (...) I think I will make a Police Report. against him* (husband). - lowers the voice - *Then she said, I'll give you some advice, don't do it!* - returns to the tone of the voice - *Don't do it, because* - raises the voice - *I'm in the middle...* - returns to the tone of the voice - *they'll treat you badly and something else* [name of ex-husband] *will be angry with you and then he will kill you. We will be paying, and* - raises the voice - *The Lord* - returns to the tone of voice - *is going to solve that situation. Then, I said okay.* - lowers the voice - *I don't want his evil. (...)* (Maria da Penha, indifferent, shakes her shoulders).

## Discussion

The first axis was entitled "Women with Leprosy: Crossroads between the Skin, the Shape and the Feminine" and, in it, we approach the concerns of women in relation to the female body and appearance when affected by leprosy.

A territory of concerns opens up for them; and everyday issues, such as how to dress, the possibility that the others will see the spots and leprosy, gradually shape the meetings. This same territory, from the experience with leprosy, could open itself to questioning social impositions as to how to follow a certain female model.

The society where we live encourages a close eye on body aesthetics, in most women. Such evidence is in magazines, television, encouraging and showing that there is a beauty standard^2^. Power relations are marked by discipline. In this sense, the bodies shown here are disciplined by a "normative imposition" in a manufacturing process of individuals^17^. Society establishes the doctrines to be followed, and the designed body is affected by that society in which it is inserted^2^.

An international research study^18^ refers to body image as being linked to a mental concept but, above all, it is related to a social phenomenon. It points out that factors such as Body Mass Index (BMI), social media, self-esteem, and family relationships, exert pressure on the individuals and especially on women.

One of the interviewees said, "I stopped caring about my looks these days," considering that I wasn't cutting my nails or dyeing my hair. We can think of beauty as an institution, as well as health and language, among many others. Others had transformations in their appearance, such as hair loss and changes in the way of dressing, but they reinvented themselves in the face of difficulties, as can be seen in the "put on some hair extension" statement. 

With the autonomy of women taking force and being present in several spheres, the historical system of domination over the woman's body had rearrangements, creating new ways of maintaining domination over the subjectivity of bodies, one of them through the fixation of beauty for a certain aesthetic standardization^19^. For researchers, violence and domination of the female body are present and intensified today and reinforce the hierarchy between genders^20^^-^ 21. 

Other authors have also noticed shame and suffering in the report of women affected by leprosy due to transformations in their bodies, such as spots and functional transformations^2^. Leprosy and its treatment will override the established way of being a woman, with a given appearance, a given weight, a given skin color. Another overlap is made between the prejudices triggered by the black skin color (transformation caused by the treatment) and the disease, as in "the drug made me black".

The Brazilian history is composed of centuries of black slavery and the imposition of white domination^22^. This subordination left marks in the social imagination and is expressed in the rejection of the possibility of having a blackened skin and in the negation of the black in us. Thus, women with leprosy being treated and with darkened skin gradually overlaps conditions of social non-acceptance^23^.

The authors^24^ bring us the idea of serialized subjectivity, a certain way of being and being in life that befits integrated world capitalism. In this sense, women in the instituted way must be vain, take care of their appearance, have no spots, be thin, adding up to a series of impositions that are sometimes incompatible with being affected by leprosy or even with the condition of life, including genetics. In this way, health professionals could adopt active listening to these aspects that generate suffering and isolation. The encounter between the health professional and the woman with leprosy could constitute a field for the movement of life power.

Every individual is on a plane of consistency, in which they become variable at all times, affecting each other, according to the relationship that is built, in order to develop a degree of power, a power to be affected^25^. We can say that the meetings between professionals and users are not always powerful. Very often, the clinical reasoning centered on the biological body does not consider these aspects and naturalizes the transformations of the body, sometimes for women, a new body. A study^26^ points out that, even with the determination of the cure, leprosy can still be present in the body marks, which can lead women to change the way they see their bodies and change the treatment given to it, in a negative way, relating the new body with the disease and remembering the past body with a healthy life. This shows us that, even with the cure, the lives of these women can remain marked, requiring a reformulation in the way of life.

In this way, apathy and rejection find their way into the lives of these women, although other ways of living are possible, other transformations. The transformations are made up of escape lines, among which there is the becoming-a-woman and so many others. Of these, the essences and meanings unfold according to a more intensive element, in which affections are modulated^27^. The becoming-a-woman has no need to enter into the policies of multiculturalism or gender policies; after all, this transformation can generate new subjectivities that have not yet been taken by consumerist capitalism, nor by Christian morality^27^.

In the second axis, the participants brought anguish and concerns about remuneration for maintaining life. The work situations of these women were also observed. There is a new overlap between social status and leprosy, between jobs performed and wages, between low schooling and the profession, between disrespected labor rights and the obligation of domestic work.

Corroborating other studies^6^^,^ 28, we can also observe that the schooling of the majority did not exceed high school and, also, the work activities did not require specific training. As a consequence, we find informal work contracts, generating absence of benefits, such as sickness benefit and paid leave^28^. Concomitant to this, it is reasserted that low schooling can favor losses related to precarious living and health conditions, aggravating vulnerability to diseases and poverty^29^^-^ 30.

Considered a neglected disease, leprosy invites us to reflect on elements intrinsic to this, such as, for example, the relationship between the involvement of low-income people and the low interest of the technology, equipment and pharmaceutical industries in the development of measures to solve the problem^6^^,^ 30. This great relationship between the disease and low income indicates that there can be an association with the social determinants, since leprosy is found in areas with greater pockets of poverty^30^.

Some interviewees sought ways to guarantee sustenance and also had social services, in addition to the unit where they conduct the treatment, to help with provisions for the home: "I get the (basic) basket" and "I get help from the social worker". A new overlap occurs: unfavorable economic condition, female condition and leprosy. Leprosy is classified as a public health problem, due to its ability to bring physical, social and economic disabilities^31^. People affected by leprosy with lower incomes and lower schooling levels are susceptible to developing physical disabilities, since there are precarious living conditions and obstacles to accessing the health service^30^. When the diagnosis is late, the quality of life of the affected individuals can change, in view of the potential of leprosy to damage to the nerves^32^.

One of our interviewees appears to have some involvement of the nerves in her arms, which makes it difficult to do the job, but does not prevent her from doing it: "Not even if it hurts, you must keep on going". With access to work in not well-paid professions, the women interviewed reported experiences of dismissal, loss of rights. From the working conditions, precarious living conditions emerge, since the sum of time of private and formal domestic service, in addition to the lack of time to sleep, generates physical and mental illnesses^33^. The authors^34^ assert that the increase in jobs in situations of precariousness is due to the growth of salaried jobs without a formal contract and of self-employment. This can culminate in the violation of rights if the person needs some benefit. In addition, women raised concerns about retirement and the maintenance of life in their old age.

Sometimes, health professionals carry out educational actions to tell users about the disease, its impacts and treatment. With the theoretical framework that supports this study, we assert that health education carried out in the instituted manner reproduces logics and does not change what is happening, as it is based on the idea that people freely choose their ways of living, choosing for example to eat healthily, practice sports etc., and that, for such, it is enough to be informed by some specialist to change the behavior. We believe that people from disadvantaged social classes will eat what they can, work as they can and survive under the circumstances imposed by their social conditions.

Returning to the idea of transformation, we bet on education as a meeting power for multiple transformations, a triggering of powers, giving possibility for the creation of other lines, with the opening of other paths, managing the small openings in the active organizations^35^. The experience of the disease could produce new transformations and reflections on, for example, the domestic work as women's attribution. Some reported adaptations, for example, "pots, I don't polish anymore", although they didn't stop washing them. A study^36^ states that the more a culture aims at women within the domestic sphere, the less that group will have opportunities to achieve equality in any sphere, with respect to men. In the form of a manifesto, there is the struggle of women for a more just and egalitarian society, whose representative is the feminist movement^37^.

In the "State Violence in the Professional Practices" axis, we saw situations in which the State, which manifests itself in the professional practices, in this case professional medical and police practices, discourages the struggle for access to women's rights. We consider that the professional practices are not just a set of techniques and knowledge, they are not neutral practices, but political actions that carry ideological positions, not assumed as such. Thus, it is through the professional practices that there is materialization of state violence, which we consider to be unacceptable and a cause of exclusion and repeated violence against women. 

These practices are linked to historical aspects and, in this sense, it is worth remembering that the history of leprosy is strongly marked by the presence of the State, determining who should be isolated. Such forms of treatment were established according to the power lines of the respective historical-cultural moment^38^. These attitudes culminated in violent practices to those involved until today, inviting us to reflect on the policies already established and those to come^39^^-^ 40. This context shows the way in which social constructions, such as those focused on body and gender standards, mark the lives of people with leprosy. Therefore, the production of their care requires the consideration of factors such as socioeconomic, cultural, body changes, sexuality and gender. The participants reported losing their jobs due to the disease. One of them reports that she talked to the physician, seeking some support regarding the loss of her job; however, the professional convinced her otherwise. This indicates that the actions of the State regulate the lives and rights of people affected by leprosy. Another participant brought an emotional account of a life of violence caused by her ex-husband. Domestic violence against women contains many factors, but it is a problem rooted in power relations, as a consequence of gender inequalities^41^. 

State violence is demonstrated by making it difficult to guarantee access to rights and preventing access to the justice system^42^. As we have seen, the police contradict the desire of Maria da Penha who wants to report her husband, at the time, for assault. People affected by leprosy generally have low incomes, experiencing daily violations of their rights. Against this background, it is observed that there is little knowledge about their social security and assistance rights, and there is not always guidance from the health professionals^43^^-^ 44. Sickness benefit and retirement are social rights conquered and should be accessible to everyone; however, impossibilities are faced daily by the administrative power, even when there are records on the precision of law^43^. The guarantee and the use of the benefits offered by the State can improve the living conditions of the person affected by leprosy^44^. However, we consider that tackling social inequality cannot be restricted to simple aids with a basic food basket.

The history of leprosy has progressed in many ways, but there is a need for legislative progress as well. It is necessary to guarantee the rights of the current victims, so that history does not reproduce, in order to maintain inadequate conditions. We could not fail to report concerns about the current policies in the country on easing labor rights, recrudescence of retirement rules (with a social security reform that penalizes women in particular) and unfinancing of public policies such as the SUS, not only thinking about Brazilian women with leprosy, but in all the people exposed to diseases and bacteria, as well as many neglects, machismo and inequalities.

The main contribution of the study is to offer reflections on the female condition and leprosy. Another contribution, no less important, is the approach of the theme based on the theoretical and methodological framework, which can generate contributions for the care of women with leprosy.

As for the limitations, we point out that, during data production, the care provided by the nursing team was considered, especially the pre- and post-nursing consultations, but the care provided by the medical team was not considered. We consider that these aspects have not been deepened and that they could bring analytical elements to understand the situation of women with leprosy and their follow-up. For this reason, we recommend future studies on the theme of women and leprosy that focus on the relationships between professionals and women, deepening the reflections produced in this research.

## Conclusion

Leprosy interferences in women's lives are shown in the relationship with their bodies that are already socially modeled, in the relationship with domestic work and with the means for support. There is an overlap and interference of the female condition in a patriarchal society with aspects of the history of leprosy and the prejudice that still accompanies it.

The findings show the violations of rights and the ways in which these women have dealt with them, undergoing reformulations and reinventions. Those are resistance gestures translated into continuity of actions that ensure daily life and, thus, new ways of living are being produced, in transformations and facing leprosy. The reinvention processes permeate the creation of their reception networks, the maintenance of their own life projects, not giving up facing limiting contexts, the persistence in the continuity of the treatment of the disease, and the effort in the search to overcome life's traumas. We bet on the strength of the becoming-a-woman, inviting health professionals to consider singularities and the context, in order to produce services that are meetings to assert the power of life.
